# Effects of Three Sludge Products from Co-Treatment of Wastewater on the Soil Properties and Plant Growth of Silty Loam

**DOI:** 10.3390/ijerph19074385

**Published:** 2022-04-06

**Authors:** Degang Ma, Yuxin Wang, Yu Ye, Xiaomei Ge, Xuebin Lu

**Affiliations:** 1 School of Environmental Science and Engineering, Tianjin University, Tianjin 300354, China; yuxinw@tju.edu.cn; 2 Qingyuan Environmental Monitoring Station, Qingyuan 511500, China; 18322267906@163.com; 3 CECEP Talroad Technology Co., Ltd., Beijing 102200, China; gwennege@163.com; 4 Department of Chemistry and Environmental Science, School of Science, Tibet University, Lhasa 850000, China; xbltju@tju.edu.cn

**Keywords:** sludge biochar, silty loam, aggregate stability, heavy metal, soil improvement

## Abstract

Currently, little is known about systematic comparisons of sludge products obtained from different sludge treatment processes in terms of land use. Moreover, it is worth evaluating whether the sludge produced from the co-treatment of industrial wastewater and domestic sewage can be applied to land use. In this study, three sludge products derived from the same municipal sludge—sludge biochar (SSB), dried sludge (DSS), and sludge compost (SSC)—were added to silty loam (SL) at a 20% mass ratio to assess their effects on soil structure, properties, and fertility. Chinese cabbage was planted as a model crop and its growth and physiological state were monitored. The experimental results showed that the water retention of the soil was significantly related to its porosity, and the moisture in the three sludge products-modified soil mainly existed in the form of free water. The addition of three sludge products increased the total porosity of SL. SSC enhanced the water retention of SL by increasing the capillary porosity, and SSB improved the gas permeability of SL by increasing the non-capillary porosity. The three sludge products all increased the content of large particles in the soil and improved the stability of the aggregates of SL. Among them, SSB and DSS had significant effects on improving the stability of the aggregates. Although the addition of the three sludge products improved the fertility of SL, compared with that of DSS and SSC, the addition of SSB made the growth indices of Chinese cabbage the best, indicating that SSB can effectively maintain soil nutrients. The heavy metal test results of Ni showed that SSB had a good stabilizing effect on heavy metals. Therefore, compared with drying and composting, pyrolysis of municipal sludge is more suitable for SL improvement.

## 1. Introduction

The co-treatment of industrial wastewater and domestic sewage is a commonly used sewage treatment method, which can greatly enhance the environmental, economic and social benefits of wastewater treatment. Combined treatment after scientific planning can reduce the construction and operating costs of wastewater treatment plants and improve the treatment effect of industrial wastewater [1,2]. The sludge obtained in this mixed sewage treatment process is a type of municipal sludge, which is a kind of biosolid produced during the concentration and dehydration process of municipal sewage treatment.

Over time, landfills, incineration, land use, and other resource utilization have been explored as the main treatments and disposal methods of sewage sludge in most countries [3,4]. Sewage sludge is rich in beneficial nutrients such as nitrogen, phosphorus, potassium, organic matter, and trace elements, and can be effectively applied to soil improvement and remediation. Apart from realizing the recycling of valuable nutrients, land application of sewage sludge to contaminated soil can improve the physical and chemical properties and microbiological characteristics of the soil [5], as well as promote plant growth and productivity [6]. Nevertheless, raw sewage sludge contains harmful substances such as pathogenic bacteria, parasitic eggs, organic pollutants, and heavy metals [7]. In detail, heavy metals, pathogenic bacteria, and other harmful substances can easily infiltrate the groundwater, which triggers a series of water pollution processes. In other words, unwise application of raw sewage sludge may lead to excessive heavy metal content or other secondary pollution in the soil [8,9]. A high content of heavy metals has become an important factor hindering the land use of sewage sludge. The content of heavy metals in municipal sludge depends on the proportion of industrial wastewater and the nature of the industry. After secondary treatment of sewage, more than 50% of heavy metal in the sewage is transferred to the sludge [10]. Therefore, the content of heavy metal in municipal sludge is generally high. In China, before being used as fertilizer, municipal sludge should meet the requirements of “Pollutant Control Standard Limits for Agricultural Use of Sludge, Table 6” in “Emission Standard of Pollutants for Urban Wastewater Treatment Plants” (GB18918-2002).

Many works at home and abroad focused on the treatment and utilization of sludge, and various kinds of solutions have been proposed. For example, pyrolyzing sludge to produce biochar is an alternative method [11]. Velghe et al. found that almost all heavy metals in sewage sludge were concentrated in biochar according to low-temperature pyrolysis, and the heavy metal contents were lower than the standard application values, so the biochar could be used for the adsorption of pollutants in water [12]. The sludge pyrolysis study of Yuan et al. showed that Cu, Ni, As, Zn, Pb, and Cd were all enriched at 500 °C, 600 °C, and 700 °C, and the residual rates were high, which explained that the stability of heavy metals was better than that before pyrolysis [13]. Moreover, composting is also a feasible method [14]. As a soil amendment, sewage sludge compost may improve urban soils as it contains a variety of plant nutrients, such as nitrogen (N), phosphate (P), potassium (K), and organic matter, which further enhances soil porosity, moisture content, and soil aggregates [15,16,17]. Additionally, the positive impacts of the application of sewage sludge compost on soil properties are usually conducive to the growth and nutrient absorption of various garden plants [18]. In addition to these studies, there are a large number of studies that have evaluated the effects of the dosage and types of sewage sludge on the growth and development of different plants, and the experiments ranged from indoor to outdoor in order to explore the application value of sewage sludge [19,20].

As far as we know, the contents of heavy metals and other toxic organics of municipal sludge produced by co-treatment of mixed sewage are usually higher than those of domestic sewage sludge, which makes its application in land use restricted. With comparative analysis, we tend to adopt the method of pyrolyzing municipal wastewater sludge before land use, which can effectively decompose toxic organic matter and stabilize heavy metals. However, whether the method of pyrolysis has advantages in other aspects of soil improvement needs to be investigated. In previous studies, there has not been a systematic comparison of sludge biochar (SSB), dried sludge (DSS), and sludge compost (SSC) derived from the same type of municipal sludge. Therefore, to address the above knowledge gap, the silty loam (SL) improvement experiment of the three sludge products produced by municipal sewage treatment was carried out, and the purpose of this study was to investigate the improvement mechanisms of three municipal sludge products added to SL, and verify the effects by setting up a pot experiment with Chinese cabbage as a model crop (43 d), which could provide a scientific reference for the land use of municipal sludge.

## 2. Materials and Methods

### 2.1. Material Properties

SL refers to soil with a particle size of 0.2 mm to 0.02 mm and a texture between those of clay and sand. The SL used in the experiment was collected from the surface soil (0–20 cm) of the planting area of a factory in Dongli District, Tianjin. DSS, SSC, and SSB were taken from Qingninghou Sludge Disposal Center in Tianjin, and the SSB was obtained by pyrolysis of DSS at 490 °C. The basic physical and chemical properties of the silt loam used are shown in Table 1. The physical and chemical properties of the three sewage sludge products are shown in Table 2.

### 2.2. Preparation of the Pot Experiment

The pot experiment was carried out in Tianjin from September to October 2018, using polyethylene flower pots as planting containers (30 cm upper diameter, 25 cm lower diameter, and 20 cm height), and the experimental plant was Chinese cabbage. There were four treatment groups set up in the experiment: (1) SL (control), (2) SSB + SL, (3) DSS + SL, and (4) SSC + SL. There were three replicates in each group. The SL was sieved (<2 mm), and SSB, DSS, and SSC were mixed with the SL by a mechanical mixer at a 20% dry weight addition. (According to the preliminary exploration experiment, it was determined that adding 20% SSB to the soil had the best planting effect, so this ratio was used in the experiment). For convenience of comparison, the same proportion was used for SSC and DSS. The soil samples were mixed for 48 h to obtain homogenization. In each polyethylene pot, 50 full cabbage seeds were selected to be sprinkled evenly on the surface of the soil and covered with fine soil. The soil water content was kept between 60% and 80% of the maximum water holding capacity, and the greenhouse temperature was kept at approximately 25 °C during the day. Mature Chinese cabbage was harvested after 43 days of growth.

### 2.3. Determination of Soil Physical Properties

The cutting ring method was used to determine the moisture content, saturation moisture content, and bulk density. The total porosity was calculated by *p_t_* = (1 − *b*/D) × 100%, where *p_t_* is the total porosity, D is a constant, that is generally 2.65 g/cm^3^, and *b* is the bulk density. The capillary porosity was determined by the cutting ring method. The non-capillary porosity was calculated by *p*_0_ = *p_t_* − *p_c_*, where *p*_0_ is the non-capillary porosity, and *p_c_* is the capillary porosity. The above analysis methods were based on the series of soil testing standards “NYT 1121-2006” of China. Water-stable macroaggregates were determined by the wet sieve method. The soil mechanical composition was determined by the Malvern laser particle size analyzer [21].

### 2.4. Determination of Soil Chemical Properties

The total salt content was determined by the residue drying–weighing method. Hydrolysable nitrogen (HN) was determined by the alkali-hydrolyzed reduction diffusing method. Available phosphorus (AP) was determined by the hydrochloric acid and sulfuric acid solution leaching method. Available potassium (AK) was determined by the ammonium acetate leaching–flame photometry method. Organic matter (OM) was determined by mass loss at 550 °C ignition. The above analysis methods were based on “Chemical Analysis of Soil Agriculture” [22]. The electrical conductivity and pH value were determined by a pH meter, in a 2.5:1 water-soil ratio.

### 2.5. Heavy Metal Stability Experiment

Nickel (Ni) was evaluated as a representative heavy metal to explore the stability of heavy metals in SSB. SL was mixed with SSB and standard electrolytic nickel powder in 6 treatments: (1) SL + Ni (mass ratio 1500:1), (2) SL + SSB + Ni (mass ratio 1300:200:1), (3) SL + SSB + Ni (mass ratio 1000:500:1), (4) SL + Ni (mass ratio 1500:50), (5) SL + SSB + Ni (mass ratio 1300:200:1), and (6) SL + SSB + Ni (mass ratio 1000:500:1). Each group was repeated 3 times. Soil samples were mixed uniformly for one week, and nickel in its four states (oxidizable state, residual state, reducible state, and exchangeable state) were determined.

Determination of six heavy metals (copper, zinc, nickel, chromium, cadmium, lead) was carried out by microwave digestion and graphite furnace atomic absorption spectrophotometry.

### 2.6. Measurements of Plant Growth Indices

(1) Germination number: The germination number was determined on the 15th day after sowing.

(2) Plant height: At the end of planting, the plant height was measured from the bottom to the top with a tape measure.

(3) Aboveground/underground fresh weight: The aboveground or underground parts of the plant were rinsed with deionized water, wiped dry, and weighed to obtain fresh weight.

(4) Aboveground/underground dry weight: The aboveground or underground parts of the plant were pretreated, treated at 115 °C for 15 min, dried at 75 °C to constant weight, and weighed to obtain dry weight.

## 3. Results

This section may be divided by subheadings. It should provide a concise and precise description of the experimental results, their interpretation, as well as the experimental conclusions that can be drawn.

### 3.1. Effect of Three Sludge Products on the Chemical Properties of SL

The chemical properties of the original SL and three improved soils are summarized in Table 3. It can be seen from the table that the SL as control group was slightly alkaline (pH = 7.48), and the addition of the three sludge products effectively reduced the soil pH. Based on the fact that the electrical conductivity of the soil was positively correlated with the value of the total salt content, the addition of DSS and SSC increased the electrical conductivity of SL, which meant an increase in salinization, while the addition of SSB led to a lower electrical conductivity of the soil, as the ammonium salt volatilized during the pyrolysis and carbonization of the sewage sludge, making the total salt content in the SSB lower than that of DSS and SSC [23].

It can be seen from the table that the fertility of the original SL was poor, and the application of SSB, DSS, and SSC could effectively promote the content of organic matter (OM), hydrolysable nitrogen (HN), available phosphorus (AP), and available potassium (AK), which was increased to 1.36~1.59 times, 29.3~31.4 times, 89.5~127.4 times, and 7.21~9.54 times the original content. It is evident that SSC and SSB had a stronger impact on the improvement in soil fertility than that of DSS, and the modified soil with SSC reached maximum values of OM, AN, and AK. This was because after the composting of sewage sludge, nutrients were activated, and humus and OM increased [6]. Moreover, SSC used as substrate of plant growth could affect the activity of enzymes in the soil, as the application of sludge might increase soil nutrients and improve soil structure [24]. Adding nitrogen and phosphorus to the soil could promote hydrolytic enzyme activities, such as urease and phosphatase, which led to the increase in soil fertility [25].

### 3.2. Effect of Three Sludge Products on the Physical Properties of SL

(1) Water retention capacity and porosity

**Figure 1:**
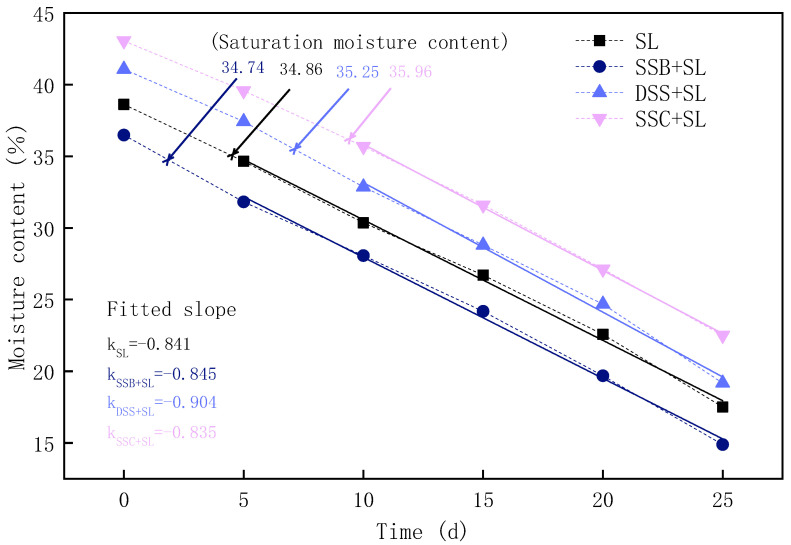
Changes in moisture content of four soil samples (SL, SSB + SL, DSS + SL, SSC + SL).

Figure 1 depicts the water retention capacity of the four groups of soil samples. The saturated water content of SL, SSC-modified soil, DSS-modified soil, and SSB-modified soil were 34.86%, 35.96%, 35.25%, and 34.74%, respectively, indicating that the addition of SSC and DSS increased the saturated water content of SL, while the addition of SSB slightly reduced it. In order to observe the combination state of water in soil, the starting point in Figure 1 was the supersaturated water content state. It was found that when the water content decreased to the saturated value, the curve adhered to the rate of decrease before the saturated water content, which was a linear relationship with time. It showed that the moisture in the soil mainly existed in the form of free water, and the water evaporation characteristics were consistent with that of liquid surface evaporation. A linear fit was performed on the points whose values were less than the saturated water content in the curve, and the slope obtained represented the water holding capacity of the soil. As shown in Figure 1, the slopes of the water retention curves of the four groups of soil are k_SL_ = −0.841, k_SSB+SL_ = −0.845, k_DSS+SL_ = −0.904, and k_SSC+SL_ = −0.835. This result showed that the addition of SSC reduced the loss rate of the soil moisture content; in other words, it improved the water retention performance of SL. The addition of DSS and SSB slightly reduced the water retention of SL, which was mainly related to the porosity of the soil and the hydrophilic properties of the particle surface. Furthermore, the influences of biochar on soil water holding capacity were unequal due to different factors such as soil texture and biochar type [26]. In line with that, TRYON et al., (1948) found that influences on available water of soils with different textures by adding biochar varied greatly, and the results of this research demonstrated that biochar could significantly increase the available water of AL (approximately an 18% increase), did not excessively affect the available water of SL, and had a reduced impact on that of clay soil [27]. This was in accordance with the research of Xu et al., who indicated that biochar could be applied to improve the water retention capacity only in coarse soils or soils with large pores [28]. Therefore, in this study, the improvement effect of SSB on soil water retention was not as significant as that of SSC and DSS, which was determined by the porosity and hydrophilicity of the sludge products and SL.

**Figure 2:**
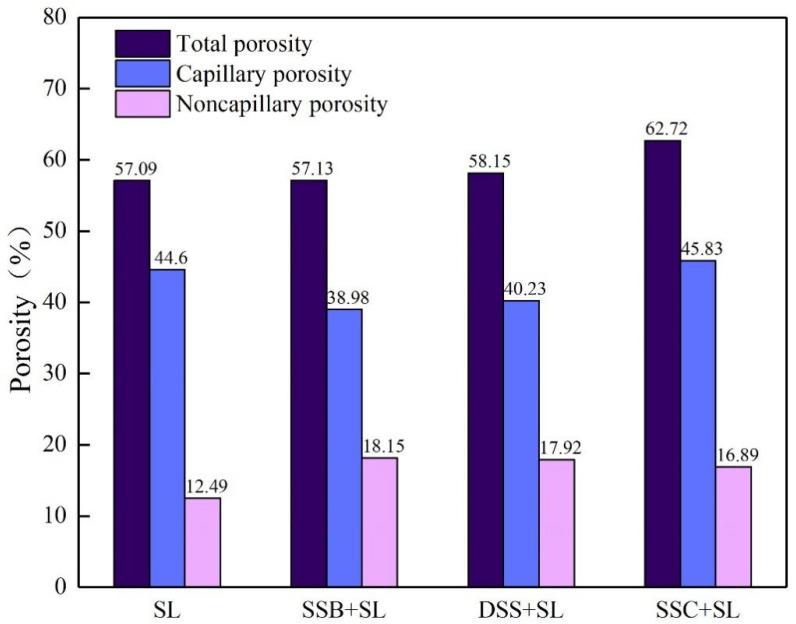
Comparison of three porosities (total porosity, capillary porosity and non-capillary porosity) for four different samples (SL, SSB + SL, DSS + SL, SSC + SL).

As shown in Figure 2, compared with the control group, the porosity of the three groups of modified soil changed to some extent. The total porosity of the SSC-modified soil increased slightly and mostly increased by non-capillary pores. The total porosity of the DSS-modified soil and SSB-modified soil did not change much, but compared with that of control loam, the capillary porosity decreased while the non-capillary porosity increased. Under natural conditions, when the pores between soil particles (including capillary pores and non-capillary pores) are filled with water, the moisture content is at what is called the saturated moisture content, which could be increased by adding SSC and DSS. The application of SSB reduced the saturated moisture content of the soil (Figure 1), Moreover, compared with the capillary porosity of the original SL (44.6%), the addition of SSB and DSS reduced the capillary porosity, and the addition of SSC increased the capillary porosity of SL and thus improved the water retention of SL, which was consistent with the results of four groups of fitting slopes of the water retention curve. This might be the result of the small total porosity value, especially the capillary porosity value of the SSB-modified soil and DSS-modified soil, resulting in the reduced water retention capacity. The SSC-modified soil had the ability to hold more water than the control soil, which was attributed to its higher total porosity and capillary porosity. This result showed that the water retention of soil had a high degree of correlation with its porosity. The increase in total porosity could lead to a growth of saturated water content, while increasing capillary porosity could keep the water in the soil in the capillary pores, thereby improving the water-holding capacity of the soil. In addition, in view of the fact that the non-capillary porosity affected the gas permeability of the soil [29], the addition of SSB increased the non-capillary porosity and the addition of SSC and DSS reduced the non-capillary porosity, indicating that the addition of SSB enhanced the gas permeability of the soil and facilitated the exchange of gas, which had a positive impact on crop growth.

(2) Soil aggregates and mechanical composition

**Figure 3:**
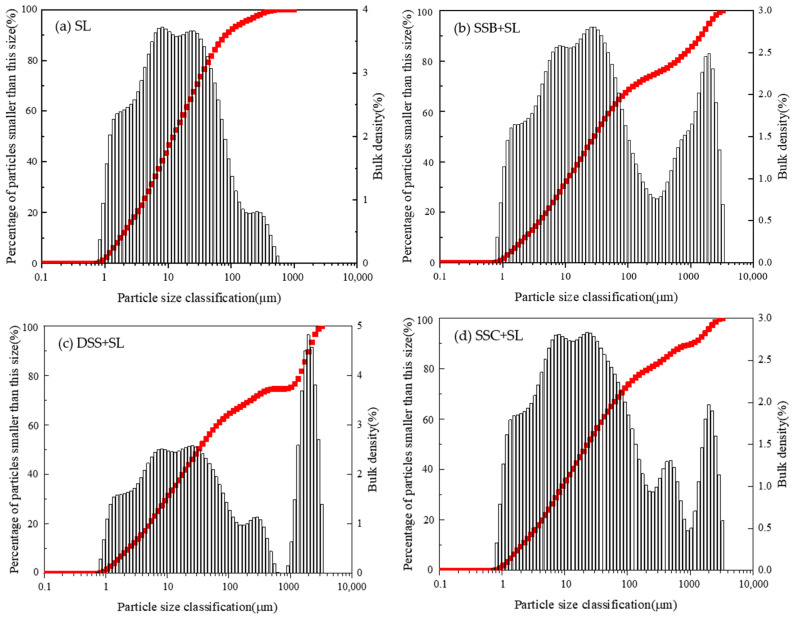
The particle gradation curve of four different samples.

The particle gradation curve of the soil is shown in Figure 3. It can be seen from Figure 3a that the particle distribution of SL was concentrated in the range of 2–100 μm, and the overall distribution was relatively uniform. In Figure 3b, the particle gradation curve of the SSB-modified soil was relatively smooth, and there were two peaks. Compared with Figure 3a, it can be seen that the addition of SSB increased the particulate matter of more than 1000 μm, which may be because the agglomeration of SSB in the water environment made the particles suspended in the solution larger and the measured value was higher. Figure 3c explains that the particle size of DSS-modified soil was more distributed around 1000 μm. This was because polyacrylamide was added in the sludge drying process to make the fine suspended particles and colloidal particles in the sludge coalesce into larger flocs. Figure 3d shows that there were multiple peaks in the particle gradation curve of SSC-modified soil. The reason might be that SSC contained sludge particles as well as a large amount of straws and other substances, and it was difficult to separate the sludge particles and light straws with the dry sieving method, which was determined under the action of hydraulic sedimentation.

Through the particle gradation curve, the percentages of clay particles, silt particles, and sand particles were calculated, and the soil texture was determined according to the soil trigonometric map. The results are shown in Table 4. The addition of three sludge products increased the sand content of SL and decreased the clay and silt content. According to international standards, the texture of the control group was silty loam, and the texture of the other three improved loams was sandy loam.

As shown in Table 4, after adding three sludge products, the content of macroaggregates in the soil increased. This was because the original SL was characterized by high salt content and poor soil colloid cohesion, and excessive salinity affected the structure and activity of the microbial community as well as the accumulation of soil organic matter, which was not conducive to the formation and stability of soil aggregates [30]. The application of sludge products improved the spatial structure of the soil, thereby improving the soil aggregates. In detail, the large aggregates in the soil of the SSB group, SSC group, and DSS group increased by 2.17%, 1.11%, and 2.89%, respectively. This result was consistent with the study of F. García-Orenes et al. [31]. They found that the application of biosolids (sewage sludge) significantly increased the stable percentage of organic carbon, carbohydrates, and aggregates, and the rise in aggregate stability was related to the increase in carbohydrates in salinized soils.

The application of SSB to SL could improve the total porosity and water-stable macroaggregate content, which was also reflected in the research of Glab, T. et al. [32]. They found that applying sewage sludge and biochar to the soil improved the water retention capacity, bulk density, and porosity of soil mainly depending on the amount of biochar added. Biochar could reduce the leaching of OM by promoting the formation of soil organic–mineral complexes, improving the stability of the agglomerates [33]. The research by Shang Jie et al. showed that the addition of fruit tree trunk biochar and branch biochar significantly increased the soil moisture content and water stability of large aggregates, which improved soil stability [34]. The study of Fang Bin et al. showed that biochar could increase soil microbial biomass and biological activity, enhance soil agglomeration, and thus improve soil structure [35].

### 3.3. Effects of Three Sludge Products on Plant Growth and Development

**Figure 4:**
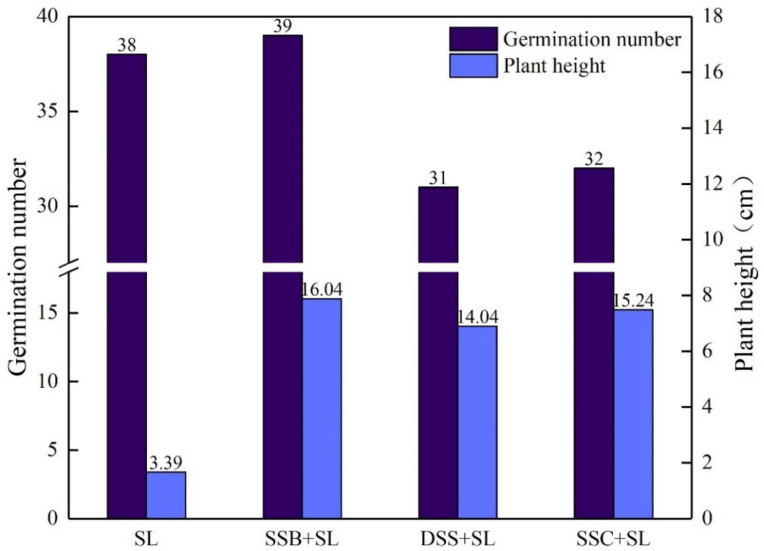
Germination rate and plant height of Chinese cabbage grown with four different sample soils.

The germination number and plant height of Chinese cabbage in the planting experiment are shown in Figure 4. Adding SSB increased the germination number of Chinese cabbage, whereas adding DSS and SSC did not enhance the germination number of Chinese cabbage, which might be because the DSS and SSC contained hormones and organic pollutants that inhibited the germination of seeds; when the addition of sewage sludge exceeded a certain amount, it inhibited the germination of Chinese cabbage [36]. Moreover, the EC of SSC and DSS were 12.59 mS·cm^−1^ and 5.37 mS·cm^−1^ (Table 2), indicating that the salt content was high, which inhibited the enzyme activity and caused damage to plants. The soil EC value should be lower than 3 mS·cm^−1^ to be suitable for plant growth [37]. Therefore, during the germination and initial growth of seedlings, SSC and DSS caused an inhibition on plant growth, while the EC of SSB was 1.68 mS·cm^−1^ (Table 2), which did not make adverse effects. Furthermore, the increase in non-capillary porosity (Figure 2) and effective decomposition or fixation of toxic substances by SSB played a significant role in promoting the germination of seeds.

**Figure 5:**
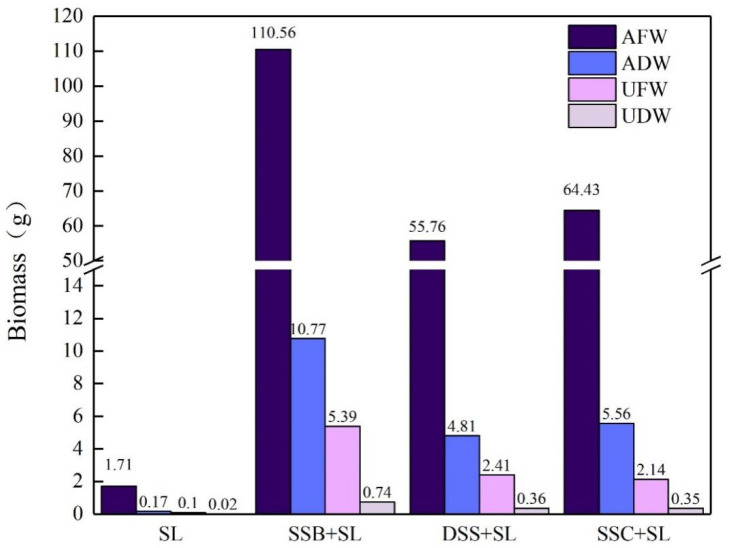
Dry and fresh weights of aboveground and underground parts of Chinese cabbage grown with four different soil samples. AFW: aboveground fresh weight; ADW: aboveground dry weight; UFW: underground fresh weight; UDW: underground dry weight.

From the perspective of plant development, the growth of Chinese cabbage in soil with SSC, DSS, and SSB was significantly more vigorous than that of the control group (plant height 3.39 cm). Among them, the plant height of Chinese cabbage planted in SSB-modified soil and SSC-modified soil was above 14 cm, and the promotion of DSS was weaker than that of the other two sludge products. The dry and fresh weights of the aboveground and underground parts of potted Chinese cabbage are shown in Figure 5. The promotion of SSB regarding the dry and fresh weights of plants was more beneficial than that of the other two modified soil groups, and these plants had the largest dry and fresh weight values (aboveground fresh weight: 110.56 g, aboveground dry weight: 10.77 g, underground fresh weight: 5.39 g, underground dry weight: 0.74 g). The addition of SSC and DSS also had a certain effect on the dry and fresh weights of Chinese cabbage, but they were not as good as SSB in promoting plant growth.

According to the results of the planting experiments and the chemical properties of the soil (Table 3), SSB could provide nutrients for SL; significantly enhance the content of soil nitrogen, phosphorus, potassium, and OM; and promote plant growth and development. As a consequence, compared with DSS and SSC, SSB as a kind of biochar could significantly enhance the exchangeable state of the main cations necessary for plant growth, and improve the availability of soil nutrients. High adsorption capacity, CEC, and chemical reactivity made it appropriately used as a fertilizer slow-release carrier, which delayed the release of fertilizer nutrients in the soil, increased the exchange and adsorption of nutrients, reduced the dissolution and migration of nutrient ions, and reduced the loss of fertilizer nutrients such as leaching. Moreover, SSB could also improve soil water and nutrients retention by increasing soil organic carbon content [38].

### 3.4. Stability of Heavy Metals in SSB

Normally, due to the limitations of industrial wastewater, mixed sewage sludge is usually more suitable for incineration treatment than land use. However, the high cost of incineration and the limited processing capacity cannot solve all the problems. For municipal sludge that meets relevant standards or is near the standard limit and has controllable toxicity, it can be used for land use after evaluation. In view of the fact that the municipal sludge contains heavy metals and toxic substances, and considering the effects of the above three municipal sludge products on soil physical and chemical properties and planting characteristics, carbonization is strongly recommended for municipal sludge co-treatment in terms of land use, and a heavy metal stability experiment was carried out in this study to verify the heavy metal fixation ability of SSB.

The results of the heavy metal stability experiment are shown in Table 5. In the six groups of treatments—(1) SL + Ni (mass ratio 1500:1), (2) SL + SSB + Ni (mass ratio 1300:200:1), (3) SL + SSB + Ni (mass ratio 1000:500:1), (4) SL + Ni (mass ratio 1500:50), (5) SL + SSB + Ni (mass ratio 1300:200:1), and (6) SL + SSB + Ni (mass ratio 1000:500:1)—the exchangeable and reducible Ni content decreased with increasing SSB addition, the oxidizable Ni content did not change much, and the residual Ni content increased with increasing SSB addition. The fact that the residual state was the most stable state indicated that the addition of SSB to the soil made the presence of heavy metals more stable.

Furthermore, using DSS as a control, the contents of six heavy metals in SSB were determined. As shown in Table 6, because the SSB was gained from the pyrolysis of DSS, in this study, only the related data of heavy metals of DSS and SSB were provided. In this experiment, heavy metal contents (except that of nickel) of the sludge products used complied with the standard limits of GB/T 23486-2009 “Disposal of sludge from municipal wastewater treatment plant-Quality of sludge used in gardens or parks (in Chinese)” (bold values in the table), and the nickel content was slightly higher than the standard value.

At the same time, a toxic leaching experiment was carried out for Ni from SSB and DSS. The preparation method of the leaching solution was conducted according to HJ/T299-2007 “Solid waste-Extraction procedure for leaching toxicity-Sulfuric acid & nitric acid method (in Chinese)”. The toxic leaching results showed that the leaching concentrations of Ni in SSB and DSS were 0.3 mg/L and 1.63 mg/L, respectively. The Ni content of SSB was higher than that of DSS (Table 6), but the amount of Ni leached from SSB was less, indicating that Ni in SSB was more stable than Ni in DSS. This result was consistent with the opinion of Méndez A. et al. [39]. They applied sewage sludge and pyrolysis-derived SSB to soil, and found that the leached concentrations of copper, nickel, and zinc in SSB-treated soil were lower than those of sewage sludge-modified soil. The research of Beesley et al. also explained that biochar could reduce the content of soluble metals, such as cadmium and zinc, in contaminated soil [40]. The main mechanism for solidifying heavy metals was adsorption rather than increasing soil pH. Therefore, SSB could reduce the leaching of heavy metals in municipal sludge and adsorb and solidify heavy metals in the soil, which was more environmentally friendly than DSS.

## 4. Conclusions

The results showed that the three sludge products—SSC, DSS, and SSB had certain improvement effects on SL, and could effectively promote the physical and chemical properties and the fertility of SL.

From the fitting slopes of the water retention curve and the porosity of four soil groups, it can be established that the moisture in the four soil groups mainly existed in the form of free water, and the moisture retention performance was related to the porosity. The addition of SSC and DSS increased the saturated water content of SL, while the addition of SSB slightly reduced that. In addition, SSC improved the water retention performance of soil by increasing capillary porosity, and SSB enhanced the gas permeability of SL by increasing non-capillary porosity. The characteristics of particle size distribution of the three sludge products increased the large particulate matter in SL, and the content of water-stable large aggregates of SL was increased by the modification of soil cohesion. SSB and DSS had better effects on improving the stability of aggregates. The planting experiment showed that DSS and SSC had an inhibitory effect on the initial growth of Chinese cabbage, while SSB could well maintain soil nutrients to promote the growth and development of Chinese cabbage. The heavy metal stability experiment results demonstrated that SSB had a strong stabilizing effect on heavy metals in the soil. Due to the limitation of heavy metals and other pollutants, it is necessary to carry out harmless and stable treatments for sludge produced by wastewater co-treatment before resource utilization. After evaluation, it is found that if this sludge is to be used for garden land use, the method of pyrolysis is more appropriate than drying and composting, which can be a feasible and effective approach to recycling and utilizing municipal sludge.

## Figures and Tables

**Table 1:** Physical and chemical properties of SL in this study.

Parameter	Value
pH	7.48
Electrical conductivity (mS·cm^−1^)	3.270
Total salt content (%)	1.0
Hydrolysable nitrogen (mg/kg)	71
Available phosphorus (mg/kg)	46
Available potassium (mg/kg)	154
Organic matter (%)	6.33
Moisture content (%)	2.33
Bulk density (g/cm^3^)	1.137
Total porosity (%)	57.09
Non-capillary porosity (%)	12.49
Saturation moisture content (%)	34.86

**Table 2:** Physical and chemical properties of three sewage sludge products.

Sludge Product	Treatment	pH	Electrical Conductivity (mS·cm^−1^)	Organic Matter (%)	TN (%)	TP (%)	TK (%)
SSC	Aerobic fermentation (55 °C)	6.76	12.59	40.57	2.99	1.43	1.01
DSS	Dehydration (105 °C)	7.22	5.37	39.40	2.05	4.09	1.13
SSB	Pyrolysis (490 °C)	7.10	1.68	24.90	2.17	5.88	1.47

**Table 3:** Chemical properties of virgin SL and soil modified with three sewage sludge products.

Soil Type	pH	Electrical Conductivity (mS·cm^−1^)	Total Salt Content (%)	OM (%)	HN (mg/kg)	AP (mg/kg)	AK (mg/kg)
SL (Control)	7.48	3.270	1.0	6.33	71	46	154
SSB + SL	7.21	2.915	1.0	8.66	2160	5860	1420
DSS + SL	7.22	3.385	1.2	9.58	2080	4120	1110
SSC + SL	7.14	4.745	2.0	10.12	2230	4920	1470

**Table 4:** Particle size distribution and texture grades of four different sample soils.

Soil Type	Water-Stable Macroaggregate (%)	Clay Content (%) <2 µm	Silt Content (%) 2–20 µm	Sand Content (%) 20–2000 µm	Soil Texture
SL (Control)	43.98	12.00	49.58	38.42	Silty loam
SSB + SL	46.15	8.50	34.81	56.69	Sandy loam
DSS + SL	46.87	8.21	33.67	58.12	Sandy loam
SSC + SL	45.09	9.49	37.77	52.74	Sandy loam

**Table 5:** Comparison of different forms of heavy metal Ni in soil samples after six groups of different treatments.

Group	Oxidizable State (mg/g)	Residual State (mg/g)	Exchangeable State (mg/g)	Reducible State (mg/g)
1	0.0540	0.4520	0.1340	0.0240
2	0.0551	0.4534	0.1304	0.0217
3	0.0588	0.4587	0.1302	0.0189
4	0.3565	29.9249	2.6140	0.4379
5	0.3556	30.2795	2.2670	0.4312
6	0.3561	30.5779	2.0440	0.3543

**Table 6:** Heavy metal contents of SSB and DSS and the standard limits of GB/T 23486-2009 “Disposal of sludge from municipal wastewater treatment plant-Quality of sludge used in gardens or parks (in Chinese)”.

	Cu (mg/kg)	Zn (mg/kg)	Cr (mg/kg)	Cd (mg/kg)	Pb (mg/kg)	Ni (mg/kg)
DSS	928	1531	367	2.6	94	207
SSB	1211	1642	378	3.5	105	226
GB/T 23486-2009 (pH ≥ 6.5)	<1500	<4000	<1000	<20	<1000	<200

## Data Availability

Not applicable.
